# Ecological factors and morphological traits are associated with repeated genomic differentiation between lake and stream stickleback

**DOI:** 10.1098/rstb.2018.0241

**Published:** 2019-06-03

**Authors:** Diana J. Rennison, Yoel E. Stuart, Daniel I. Bolnick, Catherine L. Peichel

**Affiliations:** 1 Institute of Ecology and Evolution, University of Bern, 3012 Bern, Switzerland; 2 Department of Integrative Biology, University of Texas at Austin, Austin, TX 78712, USA

**Keywords:** adaptation, parallel evolution, convergent evolution, population genomics, association mapping

## Abstract

The repeated evolution of similar phenotypes in independent populations (i.e. parallel or convergent evolution) provides an opportunity to identify genetic and ecological factors that influence the process of adaptation. Threespine stickleback fish (*Gasterosteus aculeatus*) are an excellent model for such studies, as they have repeatedly adapted to divergent habitats across the Northern hemisphere. Here, we use genomic, ecological and morphological data from 16 independent pairs of stickleback populations adapted to divergent lake and stream habitats. We combine a population genomic approach to identify regions of the genome that are likely under selection in these divergent habitats with an association mapping approach to identify regions of the genome that underlie variation in ecological factors and morphological traits. Over 37% of genomic windows are repeatedly differentiated across lake–stream pairs. Similarly, many genomic windows are associated with variation in abiotic factors, diet items and morphological phenotypes. Both the highly differentiated windows and candidate trait windows are non-randomly distributed across the genome and show some overlap. However, the overlap is not significant on a genome-wide scale. Together, our data suggest that adaptation to divergent food resources and predation regimes are drivers of differentiation in lake–stream stickleback, but that additional ecological factors are also important.

This article is part of the theme issue ‘Convergent evolution in the genomics era: new insights and directions’.

## 1. Introduction

When organisms recurrently adapt to new environments, what are the genetic and ecological factors that influence the repeatability of their evolutionary trajectories? The answers to this question should reveal the form and constancy of natural selection, as well as the constraining roles of genetic variation and gene flow. Independent, replicate pairs of populations adapting to similar ecological conditions provide a useful opportunity to address this question. Recent genetic studies of replicated phenotypic evolution have provided tantalizing clues that evolutionary trajectories may be more repeatable than previously thought [1–3]. In particular, many studies have identified regions of the genome that are repeatedly differentiated between independent population pairs adapting to divergent habitats (e.g. [4–7]). Although repeatable phenotypic divergence is generally taken as strong evidence of the role of natural selection [8–9], it is not always clear that patterns of repeated genomic divergence solely result from natural selection [10]. Furthermore, population genomic studies are usually agnostic to specific phenotypes, and most studies have not associated specific ecological factors or morphological traits with the regions of the genome that evolve consistently across replicates.

The threespine stickleback (*Gasterosteus aculeatus*) is a good system in which to quantify the repeatability of genomic differentiation and to identify the ecological conditions and phenotypic traits that are associated with regions of repeated genomic differentiation. These small fish have frequently colonized diverse freshwater habitats in the Northern Hemisphere since the retreat of the glaciers 12 000 years ago [11]. Strikingly, sticklebacks living in similar habitats often evolve similar phenotypes, suggesting that phenotypic shifts are adaptive. The availability of a number of genetic resources including an assembled and annotated genome [4] facilitates the identification of the genetic basis of putatively adaptive phenotypes [12]. Thus, this system provides an opportunity to ask whether the same genomic regions underlie evolutionary change in similar habitats and to further ask whether these seemingly adaptive regions are associated with particular biotic and abiotic environmental factors, or with specific organismal phenotypes.

One widespread example of repeated phenotypic divergence is found among pairs of stickleback populations inhabiting lakes versus streams. Lake ecotypes are adapted to feeding on zooplankton, while stream ecotypes are adapted to feeding on macro-invertebrates [13–14]. Lake–stream pairs can show considerable genetic and morphological divergence [15–18], and at least some phenotypic differences between the ecotypes are heritable [19–23]. These ‘lake–stream’ ecotype pairs have been extensively studied in Canada and Europe and show repeated phenotypic evolution [24]; i.e. lake fish from Europe and Canada resemble each other more in multiple key traits than their more closely related stream fish [25–26]. Previous work on this system has shown that parallel evolution (here termed ‘repeatability’) is imperfect, but that deviations from parallelism can be partially explained. Specifically, the degree of phenotypic parallelism is positively correlated with the degree of environmental parallelism [24]. This correlation suggests that evolutionary repeatability is indeed adaptive to some extent in this system, but that deviations from repeatable lake–stream divergence can also be attributed to adaptation to differences in ecology among lakes or among streams. This incomplete parallelism means that habitat categories (lake versus stream), environmental variables and fish morphological traits are decoupled enough to allow meaningful genome-wide association studies.

In this study, we combine genomic, ecological and morphological data from 16 population pairs of lake–stream stickleback sampled from independent watersheds on Vancouver Island, Canada, to ask three sets of questions: (1) For a given genomic region, what proportion of the 16 lake–stream pairs show similarly high genetic differentiation, and what proportion of the genome overall exhibits shared genetic differentiation?; (2) Which genomic locations are associated with variation in environmental factors (biotic and abiotic) and variation in morphological traits across lake and stream populations?; (3) Do the genomic regions underlying these traits co-localize to genomic locations that are repeatedly differentiated? If so, is there enrichment of particular categories of traits within the repeatedly differentiated regions?

## 2. Material and methods

### (a) Quantification of repeated genomic differentiation

Illumina sequence data for pairs of lake and stream stickleback from 16 independent watersheds on Vancouver Island, Canada (32 total populations) was previously generated using the double-digest restriction-site-associated DNA-sequencing (double-digest RAD) method [27], with 24 individuals sequenced from each population. Single nucleotide polymorphisms (SNPs) were identified using a standard, reference-based bioinformatics pipeline (see [24] for full details of these data); alignment of reads was done to the Jones *et al.* [4] genome assembly. For each individual, a site was only included if the read coverage was between 8 and 100. SNPs mapping to the mitochondrial DNA or unassembled regions of the genome were excluded from further analysis. Weir–Cockerham Fixation index (*F*_ST_) [28] was used to estimate genetic differentiation between each pair of lake–stream populations, then averaged over 50 kilobase pair (kbp) windows (electronic supplementary material, figure S1). These windows were constrained to have the same size and genomic locations for all lake–stream comparisons. Window-averaged *F*_ST_ values were calculated by dividing the sum of the numerators of all SNP-wise *F*_ST_ estimates within a given window by the sum of their denominators. For downstream analysis, we required that each window contained at least three variable sites.

Genomic windows were classified as ‘outliers’ or ‘non-outliers’ based on their mean *F*_ST_. We classified outlier windows as those with mean *F*_ST_ values falling within the top 5% of the genome-wide *F*_ST_ distribution within a given lake–stream comparison. Outlier classification was performed using custom R scripts. Read coverage did not differ significantly between windows classified as outliers and those classified as non-outliers for any of the 16 population pairs (data not shown).

To identify the genomic regions (windows) that had repeatedly differentiated between independently derived lake–stream pairs, the outlier windows in each single lake–stream comparison were compared across all 16 population pairs. Repeatability was estimated window-by-window as the proportion of population pairs that had a given outlier, using the following equation:

$$\mathrm{repeatability}=\;\frac{k\;*\;(k-1)/2}{n\;*\;(n-1)/2}$$

where *k* is the number of population pairs with an outlier for a given window and *n* is the number of population pairs with data for that window.

To test whether the level of repeatability was greater than that expected by chance, we ran a permutation with 10 000 iterations. For each iteration, the outlier status of a given window was randomly shuffled among the 16 population pairs and the magnitude of repeatability was re-estimated. Missing data were held in place during resampling so that the total number of windows with data for a given population pair remained the same for all iterations. These 10 000 iterations yielded a null distribution of repeatability for each window, and empirical estimates were compared against these nulls to determine statistical significance. The resulting *p*-values were corrected for multiple testing using the *p.adjust* function with the BH (alias fdr) method in R [29].

### (b) Association mapping with Bayenv

The same SNP data outlined above were used to identify genomic loci associated with variation in abiotic factors (*n* = 5), diet items (*n* = 45) or morphological phenotypes (*n* = 34) across the 32 freshwater populations from 16 watersheds (see electronic supplementary material, table S1 for a full list of traits). Data for individual traits in each of these three categories were all previously reported, with full details on the method of measurement provided in Stuart *et al*. [24].

Bayen v2.0 [30] was used to detect statistical associations between individual SNPs and each trait. SNPs with high linkage disequilibrium (*r*^2^ above 0.2) were identified using SNPrelate [31] and removed from the dataset, which left 11 440 unlinked SNPs. Only these unlinked SNPs were used to estimate the covariance matrix in Bayen v2.0 with 10 000 iterations [30]. This covariance matrix was then used in the association mapping models to account for population structure/relatedness. Population-level allele frequencies were estimated for all SNPs (68 677) and formatted as a POPfile using a custom PERL script. Average values for each morphological, diet and abiotic trait were estimated for each population and normalized by subtracting the among-population mean from each estimate and dividing by Coop *et al*. the among-population standard deviation, as suggested by Coop *et al.* [30]; this was the ENVfile. These average allele frequency (POPfile) and trait estimates (ENVfiles), along with the covariance matrix, were the input files for Bayenv. Bayenv v2.0 was run independently five times, following the methods of Blair *et al.* [32]. Each independent run used a unique random seed and had 10 000 iterations. Bayes factors and Spearman's *ρ* correlation coefficients were estimated for all SNPs and traits; both statistics were averaged across the five independent Bayenv runs before downstream analysis. The log Bayes factors for each trait were individually plotted against the corresponding Spearman's *ρ* values (i.e. in volcano plots); this allowed us to visually ensure that loci with large Bayes factors did not tend to have small correlation coefficients, as this would be an indicator of false positives.

The SNPs from the Bayenv analysis were classified as significant candidates for explaining variance in a trait if they met the following criteria: both the log Bayes factor value and Spearman's *ρ* fell in or above the 99.9th quantile of their respective distributions (see electronic supplementary material, table S1 for the Bayes factor and Spearman's *ρ* correlation coefficient significance thresholds for each trait). If a given 50 kbp window contained one or more of these candidate trait SNPs, it was defined as a ‘candidate trait window’.

### (c) Permutation tests to quantify co-localization and enrichment of candidate trait windows and repeatedly differentiated genomic windows

For all permutation tests described below, we ran 10 000 iterations to generate the null distribution, against which empirical estimates were compared to determine statistical significance.

First, we tested whether the number of candidate trait windows on each chromosome was greater than expected by chance. In each iteration, the candidate status of a window (i.e. candidate containing or not) was randomly shuffled among the 21 chromosomes and the total number of candidate trait windows per chromosome was re-estimated. Three permutation tests were run, one for each category of the trait (abiotic, diet or morphology). The same method was used to test for enrichment of repeatedly differentiated windows, with repeatability status of a window randomly shuffled.

Second, we tested whether more traits within each category type (i.e. morphological, abiotic or diet) mapped to a given window than expected by chance. For each iteration, the presence or absence of a candidate for each trait within a category was randomly shuffled among the genomic windows and the total number of mapped traits per window was re-estimated. In this permutation, the resulting *p*-values were corrected for multiple testing using the *p.adjust* function and BH (alias fdr) method in R [29].

Third, we tested whether there were more windows that were repeatedly differentiated *and* classified as candidate trait windows than expected by chance. To accomplish this test, we randomized the candidate trait windows and separately randomized the windows that were repeatedly differentiated. Here, any window shared by at least two pairs of populations was coded as repeatedly differentiated, although the results were the same if only the significantly repeatedly differentiated windows (i.e. in at least three pairs; see Results) were coded in this way. For each iteration, we re-quantified the total number of windows that were repeatedly differentiated and contained a candidate trait locus.

Fourth, this same permutation structure was used to test whether there was more overlap between the candidate trait windows of the *different* categories (e.g. candidates for diet *and* morphology) than expected by chance. In these tests, the candidate trait windows in each category were individually randomized and the total number of windows containing candidates for both trait categories was re-estimated.

## 3. Results

### (a) Repeatedly differentiated genomic windows

**Figure 1.**
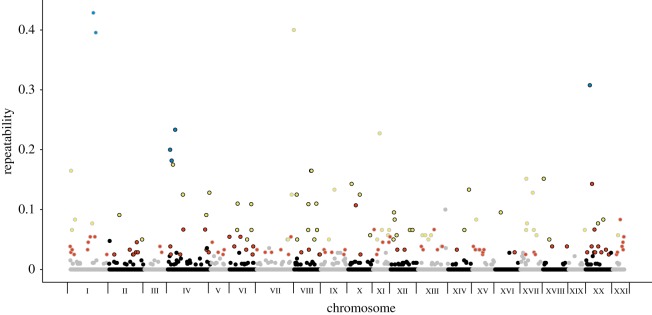
Magnitude of repeatability across genomic windows. Each dot represents a 50 kbp window; alternating grey and black lines around the dots indicate separate chromosomes. Red coloration within the dot indicates outliers that were shared across three population pairs, yellow indicates outliers that were shared across four to six population pairs and blue indicates outliers that were shared across 7–10 population pairs. Grey or black coloration within the dot indicates outliers that were either unique to a single population pair or only shared between two population pairs.

**Figure 2.**
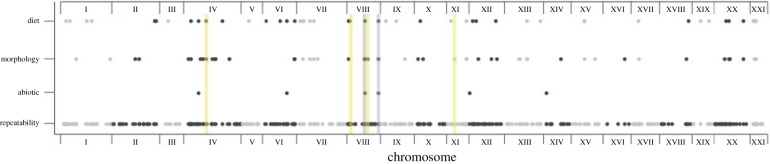
Genomic locations of the subset of candidate trait windows that map to repeatedly differentiated windows (377 total) are shown for each trait category. Each dot represents a 50 kbp window; alternating colours indicate separate chromosomes. Grey shading indicates the two windows on chromosome VIII where all three categories of traits mapped. Yellow shading indicates the four windows that contained candidate regions and were shared by the most population pairs.

Of the 2513 windows (50 kbp) across the stickleback genome, 1013 windows were highly differentiated (i.e. contained *F*_ST_ outliers) in at least one lake–stream comparison. Across the genome, 377 windows (15% of the 2513 total genomic windows or 37% of the 1013 highly differentiated windows) were outliers in two or more population pairs, indicating that there is some evolutionary repeatability at the genomic level (figure 1). Permutation testing revealed that 42 of these windows (approx. 2% of all windows, 4% of highly differentiated windows) were significantly repeatedly differentiated (*p* < 0.05) even after correction for false discovery rate. These significant windows were all outliers in multiple watersheds (a minimum of three lake–stream population pairs, a maximum of 10 out of 15 pairs with; figure 1). It is also worth noting that an additional 126 windows were outliers in three to five population pairs but did not meet the significance threshold after false discovery correction in the permutation testing (see figures 1 and 2 and electronic supplementary material, table S2 for the genomic locations of these windows). There was no difference between the 42 windows that were significantly repeatedly differentiated windows and the remaining highly differentiated windows in either recombination rate (difference in recombination rate = 1.50, *T*_475_ = 0.73, *p* = 0.467) or gene density (difference in gene density = 0.019, *T*_1448_ = 0.05, *p* = 0.69).

The outlier window shared by 10 populations was located on chromosome I. There was significant enrichment (*p* < 0.05 in a permutation test; see electronic supplementary material, table S3 for individual chromosomal *p*-values) of repeatedly differentiated windows on chromosomes VIII, XI and XXI. The location of this highly repeatable window on chromosome XXI is biologically reasonable given previous work has indicated the clustering of quantitative trait loci (QTL) on this chromosome [12]. However, very few ecologically relevant traits had been previously mapped to chromosomes VIII or XI. These sites might contain genes affecting traits that differ between lake and stream sticklebacks but that have not been genetically mapped, such as parasite resistance or behaviour.

### (b) Candidate trait windows for abiotic factors, diet items and morphological phenotypes

Using a population-level association mapping approach (i.e. Bayenv), we detected windows containing at least one SNP associated with environmental variables or fish traits. Specifically, Bayenv calculates the correlation between population allele frequency and mean trait value across the 32 populations (16 lake and 16 stream), while controlling for their genetic structure. We applied this analysis to three categories of data (abiotic factors, diet items, morphological phenotypes). Electronic supplementary material, figure S2 summarizes the locations of all windows.

All five abiotic factors tested had candidate windows that significantly explained population-level variation (correlation coefficients 0.32–0.60). Salinity and dissolved oxygen had the fewest candidate windows (four each), while the other factors had 17–20 significant candidate windows. All 34 morphological phenotypes considered had multiple candidate windows significantly associated with population-level variation (correlation coefficients 0.28–0.67). Pectoral fin area had the fewest candidates (six) while other traits had 30 or more significant candidate windows. Of the 45 diet items tested, 43 had one or more candidate window(s) and some factors had up to 20–30 significant candidate windows (correlation coefficients 0.27–0.69). See electronic supplementary material, table S2 for locations and summaries of mapped windows for each trait.

### (c) Clustering of candidate trait windows across chromosomes

There was significant enrichment (*p* < 0.05 in a permutation test; see electronic supplementary material, table S3 for individual chromosomal *p*-values) of candidate windows associated with all trait categories: abiotic factors were enriched on chromosome VII, diet items were enriched on chromosomes VII and XXI, and morphological traits were enriched on chromosomes IV, VII and XX.

### (d) Clustering of traits within candidate windows

Across the genome, there was no significant co-mapping (clustering of candidate regions) of the five different abiotic factors to a given window (*p* > 0.05 for all windows). By contrast, 45 windows (1.8% of all windows) exhibited significant co-mapping of two or more of the 34 morphological phenotypes (*p* < 0.05 in a permutation test after correction for false discovery rate). Genomic windows containing candidate loci for multiple morphological traits were located primarily on chromosomes IV, VII, XII and XX. For diet, there were 14 windows (0.6%) that had significant co-mapping of candidate loci for the 45 different diet components (*p* < 0.05 in a permutation test after correction for false discovery rate); these genomic regions were located primarily on chromosomes IV, VII and XV.

**Figure 3.**
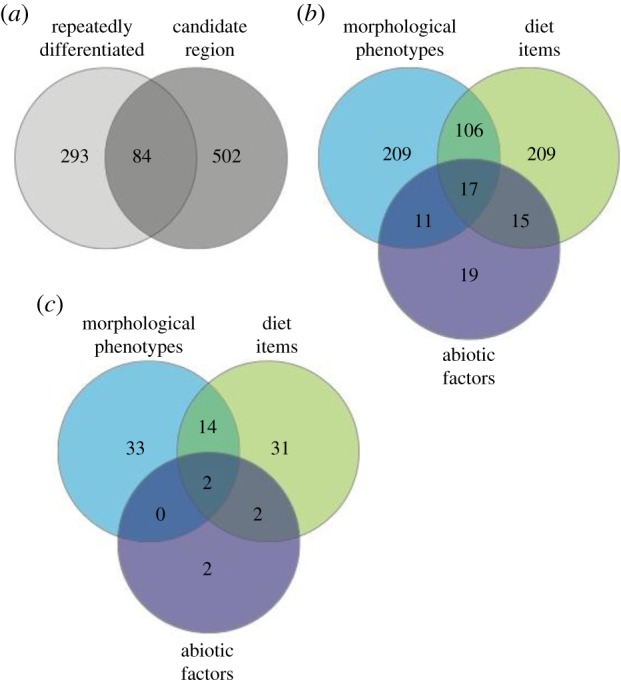
(*a*) Number of 50 kbp windows that are either repeatedly differentiated, contain candidate loci or both. (*b*) Number of 50 kbp windows containing candidate loci broken down by trait category; these are the 586 windows depicted in the darker grey circle of (*a*). (*c*) Number of repeatedly differentiated 50 kbp windows; these are the 84 windows from (*a*) that also contain candidate loci for each of the categories of traits.

Overall, there were 586 windows (23%) that contained candidate loci for at least one category of trait (figure 3*a*). Three hundred and forty-three of these windows were associated with at least one morphological phenotype, 347 were associated with at least one diet item and 62 were associated with at least one abiotic factor (figure 3*b*). There was considerable overlap of these candidate windows across the three trait categories (figure 3*b*). Correspondingly, the degree of genome-wide overlap between candidate trait windows for different trait types (i.e. diet and morphology, morphology and abiotic, diet and abiotic) was significantly more than expected by chance for all three trait combinations (*p* < 0.0001 for all three permutation tests). Seventeen windows contained candidate loci for all three categories of traits (figure 3*b*); these windows were found on chromosomes I, IV, VII, VIII, IX and XII.

When we only considered the overlap between candidate windows for diet components and the 18 morphological phenotypes that have a known role in feeding, we found that 85 of the possible 223 regions (38%) were shared (*p* < 0.0001). These windows appear to be key in determining feeding capabilities and the corresponding prevalence of prey items in the diet; they were primarily located on chromosomes IV, VII, XIII and XX, with 8–14 candidate windows on each of these chromosomes.

### (e) Co-localization of repeatedly differentiated windows and candidate trait windows

Eighty-four genomic windows (3.3%) were repeatedly differentiated (shared by two or more pairs) *and* contained a candidate locus for at least one trait (figure 2 and figure 3*a*). Six of these windows contained candidate loci for adaptation to abiotic factors, 49 windows contained candidate loci for morphological phenotypes and 49 windows contained candidate loci for diet items (figure 2 and figure 3*c*). There was an overlap of 18 of these windows among the different trait categories; two windows on chromosome VIII contained candidate loci for all three trait categories (figure 2 and figure 3*c*). However, the degree of genome-wide overlap between windows with repeated differentiation and windows containing any type of candidate loci was not greater than expected by chance (*p* > 0.05 in permutation tests for each of the three trait categories). The 40 windows displaying significantly repeatable differentiation (three or more pairs sharing an outlier window) contained candidates for between 1 and 13 individual traits (abiotic, diet or morphological). Interestingly, the four windows differentiated in the most population pairs (on chromosomes IV, VIII and XI) that also contained candidate loci were associated with diet components, swimming, feeding and armour traits (see highlighted windows in figure 2).

## 4. Discussion

In this study, we aimed to detect genomic regions with a signature of repeated differentiation across multiple independent pairs of lake–stream stickleback. We then asked whether those regions contained SNPs associated with environmental conditions and morphological divergence. Before discussing our results, we clarify that here we are not looking at parallel evolution in the strict sense, that is, whether the same mutation or allele is used repeatedly (see [33] for a discussion of different usages of the term parallel). Rather, we are interested in genomic regions of repeated differentiation. Repeated differentiation, like repeated fixation of the same mutation, strongly suggests the action of natural selection. However, it is important to note that other evolutionary forces or genomic features could also influence these patterns [10]. In our study, the repeatedly differentiated windows did not have significantly different recombination rates or gene densities from other differentiated windows, suggesting that our results are not an artefact of genome structure. Thus, finding that a region of the genome has a signature of selection (e.g. high *F*_ST_) in multiple independently derived populations implies that there may be some interesting genes or genetic features located within the region. A limitation of the reduced representation sequencing methods (double-digest RAD) employed by this study is that we are unable to distinguish between patterns of differentiation generated from direct versus linked selection [34]. Thus, we do not attempt any analyses of the specific genetic content of the genomic windows identified here.

### (a) Magnitude of repeatable genomic differentiation

Across biological systems, the degree to which differentiation is repeatable appears to be highly variable and differs depending on the biological level (gene versus phenotype versus genomic region) being considered (reviewed by [33]). Previous work in threespine stickleback has shown highly repeatable patterns of differentiation at particular candidate loci, for example, at the *Ectodysplasin* (*Eda*) gene, which has been directly shown to underlie the reduction of lateral plating in numerous freshwater populations relative to their marine ancestors [35]. A pattern of high repeatability has also been shown for the sodium/potassium ATPase (*ATP1a1*) gene, which mediates salinity tolerance [4,36].

Recent work characterizing genome-wide patterns of repeatability in sticklebacks has generally found that a small to moderate fraction of highly differentiated loci/regions evolve in a repeatable fashion [4,37–39]. Among three independent benthic–limnetic ecotype pairs of stickleback from Canada, 33% of outlier markers (SNPs) were shared by two or more of the pairs [36]; this is similar to the 37% seen for our North American lake–stream pairs. This is also similar to the 23.2% of outliers differentiating in parallel in the European anchovy (*Engraulis encrasicolus*) [7]. By contrast, for European lake–stream stickleback, only 3% of outlier windows were shared for two to four of the five surveyed population pairs [37]. This is similar to what is seen among crab and wave ecotypes of the rough periwinkle (*Littorina saxatilis*) where 3–13% of outliers are shared between at least two of the three surveyed islands [5]. In general, variable levels of repeatability for patterns of phenotypic and/or genotypic differentiation are thought to be due to a combination of environmental heterogeneity, insufficient genetic variation, variable gene flow and genetic drift [33]. Both gene flow and environmental heterogeneity influence the observed levels of phenotypic repeatability among the 16 lake–stream pairs studied here [24]. However, despite these factors, we still find a non-negligible (albeit low) level of repeatable genomic divergence for these ecotypes.

It is possible that the repeatability we observe for some genomic regions (upwards of five population pairs with shared outliers) is a signature of adaptation from standing genetic variation, as is often the case when marine sticklebacks colonize freshwater habitats [35,39–41]. Recent simulation work has shown that when populations adapt to identical environments and standing variation is present, the same alleles are most often used rather than new mutations [42]. Interestingly, Thompson *et al.* [42] also show the tendency toward repeated (parallel) evolution diminishes rapidly when selection is not entirely parallel. Indeed, Stuart *et al.* [24] have previously shown that variation in the degree of parallelism for environmental factors predicted the degree of phenotypic parallelism in the populations studied here. It could be that standing genetic variation also interacts with selective heterogeneity to produce the observed patterns.

### (b) Clustering of candidate trait windows

Many of the candidate trait windows identified in this study correspond to genomic regions previously identified as QTL for morphological, behavioural and reproductive traits (reviewed by [12]). In particular, the four chromosomes (IV, VII, XX and XXI) showing enrichment for at least one of the three trait categories (abiotic factors, diet items, morphological phenotypes) mapped in our study are among the five chromosomes previously shown to have more QTL than expected given the physical size or number of genes on the chromosome [12]. Furthermore, we found that chromosomes IV and XX were enriched for candidate trait windows associated with both feeding morphology and diet items, consistent with the previously observed enrichment of QTL associated with feeding morphology on these two chromosomes [12]. Although we did not find evidence of enrichment of candidate trait windows on chromosome XVI, the QTL enrichment on this chromosome appears to be largely driven by loci influencing body shape [12], which was not studied here. It is important to note that the work here also presents many new candidate regions that will merit future fine-scale investigation, particularly for abiotic factors and diet items, which have received relatively little attention in previous mapping studies.

It is possible that the non-random distribution of candidate trait windows is a signature of either pleiotropy or tight physical linkage. In many systems, the tight clustering of multiple loci affecting different traits (sometimes called ‘supergenes’) involved in adaptive divergence has been observed [43]. Clustering of adaptive alleles is thought to be especially important when adaptation is occurring in the presence of gene flow [44], or when it would be maladaptive to have co-adapted phenotypes broken up by recombination [45–47]. Consistent with these theoretical predictions, there is strong evidence to suggest the differentiation of the lake–stream ecotypes studied here is occurring in the presence of ongoing gene flow [24].

### (c) Association between windows of repeated genomic differentiation and candidate traits

For the candidate trait windows that were repeatedly differentiated, there was a positive, but marginally non-significant, correlation between the magnitude of repeated differentiation (number of population pairs sharing an outlier window) and the number of environmental or phenotypic traits that mapped to that window (*r* = 0.15, *F*_1,82_ = 2.88, *p* = 0.09). This suggests that loci are to some degree pleiotropic (i.e. influencing the variance of more than one trait) and may be more frequently used during adaptation to a common agent (or agents) of selection. However, such a pattern could also be generated if different agents of selection are acting in different population pairs on independent traits (and loci) that map to the same windows. Future fine-scale mapping and selection studies will be required to disentangle these alternative mechanisms.

Despite this association, we did not find evidence for significant genome-wide enrichment of candidate trait windows within regions of repeated genetic differentiation. However, we did find that all of the phenotypic traits previously identified to be highly parallel in pairs of lake–stream stickleback [24] mapped to regions of repeated genetic differentiation. This suggests that we are describing a real genetic signature of repeated phenotypic evolution. Yet, an important consideration of this study is that our use of population-level association analyses (Bayenv), rather than a within-population association study, reduced our ability to detect candidate loci. This is because the sensitivity of a population-level analysis increases when a greater number of populations exhibit the same associations between allele frequencies and phenotypes. As a result, candidate loci underlying trait variation in a single population would often be overlooked. Correspondingly, the candidate regions reported here are very likely only a subset of those important for the abiotic factors and traits considered in this study.

The observed mapping of multiple diet, feeding and armour traits to regions of the genome evolving in a repeatable fashion supports the idea that both feeding capacity and predation avoidance are among the drivers of differentiation in this system. We see a higher fraction of candidate regions related to biotic factors (predation avoidance and foraging) mapping to repeatedly differentiated genomic regions (14%) than candidate regions for abiotic factors (9%). This pattern may suggest biotic factors play a relatively greater role than abiotic factors in shaping patterns of repeated differentiation in this system. However, this pattern may also be due in part to greater variance in abiotic factors between the watersheds than between the lake and stream habitats within a watershed [24].

Despite the constraints of our methods discussed above, the repeated genomic differentiation found in this study cannot be explained solely by the variety of ecological factors and morphological traits that we considered here. This is not surprising as we know that selection in threespine stickleback is generally multifarious, involving a multitude of biotic factors such as competition [48], predation [49], parasites [50–52] and pathogens [53], as well as a variety of abiotic factors such as salinity [54], turbidity [55] and temperature [56]. Our results identify regions of the genome that are likely important for adaptation to these other environmental factors and provide a reminder that multifarious agents of selection should be considered in studies of repeated evolution. Furthermore, our results highlight the importance of integrating association mapping studies to identify links between genotypes and phenotypes with population genomic studies to identify links between genotypes and fitness. Combined, these two types of analyses can provide a more holistic view of the ecological and genetic factors that drive repeated phenotypic evolution.

## Supplementary Material

Supplementary Table 1

## Supplementary Material

Supplementary Table 2

## Supplementary Material

Supplementary Table 3

## Supplementary Material

Supplementary Figure 1

## Supplementary Material

Supplementary Figure 2

## Data accessibility

All code and input files described in the manuscript are available from the Dryad Digital Repository: https://doi.org/10.5061/dryad.pj8c6g2 [57]. The original raw sequence reads, environmental data, and SNP tables are archived by Stuart *et al.* [24].
