# Supplementary figures and images for: Ecological factors and morphological traits are associated with repeated genomic differentiation between lake and stream stickleback
Source: Philos Trans R Soc Lond B Biol Sci. 2019 Jun 3;374(1777):20180241. doi: 10.1098/rstb.2018.0241 (PMC6560272; doi:10.1098/rstb.2018.0241)

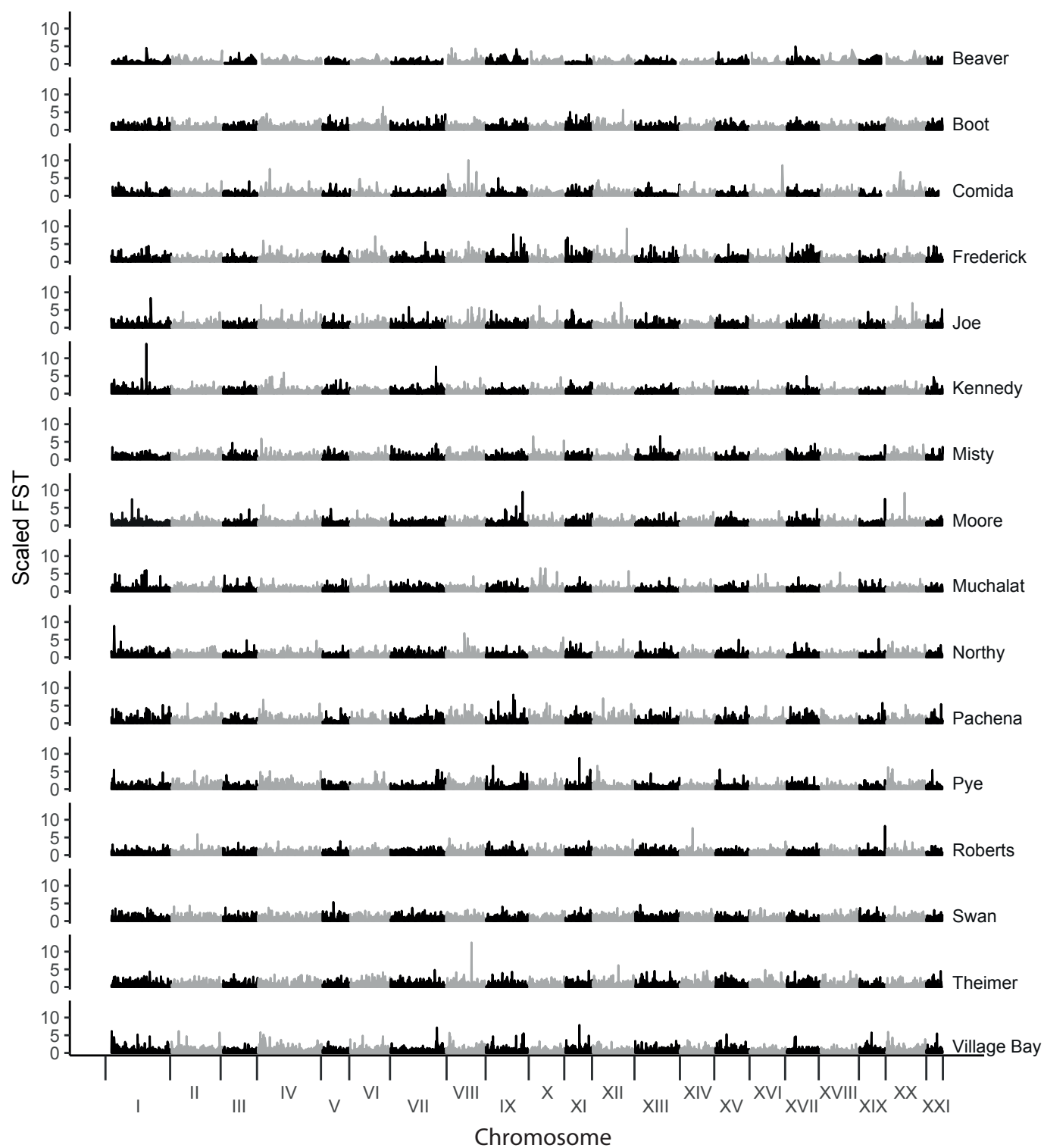

Supplement: Supplementary Figure 1 [file rstb20180241supp4.pdf]

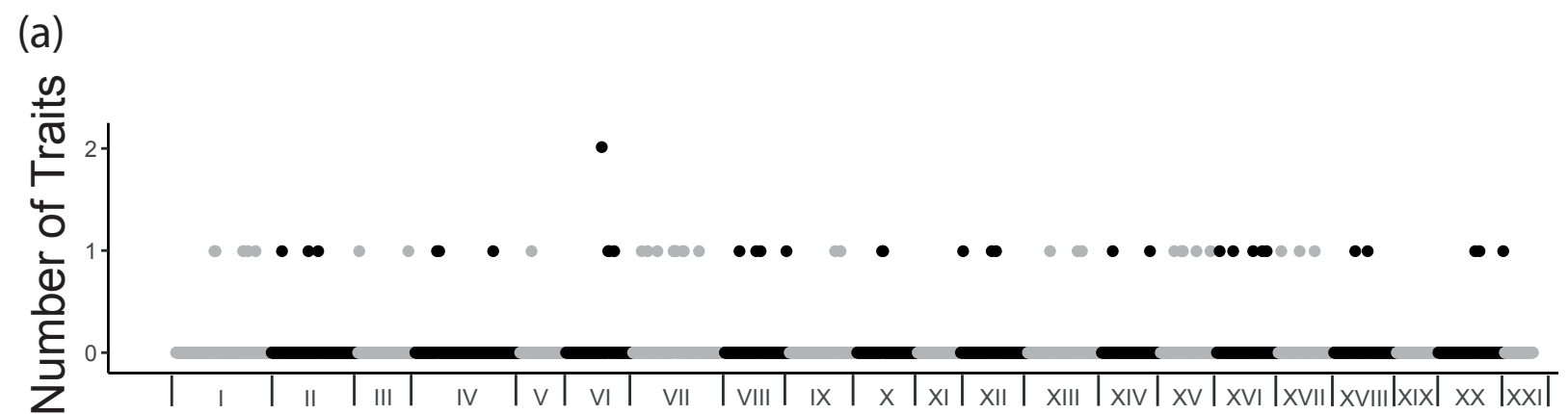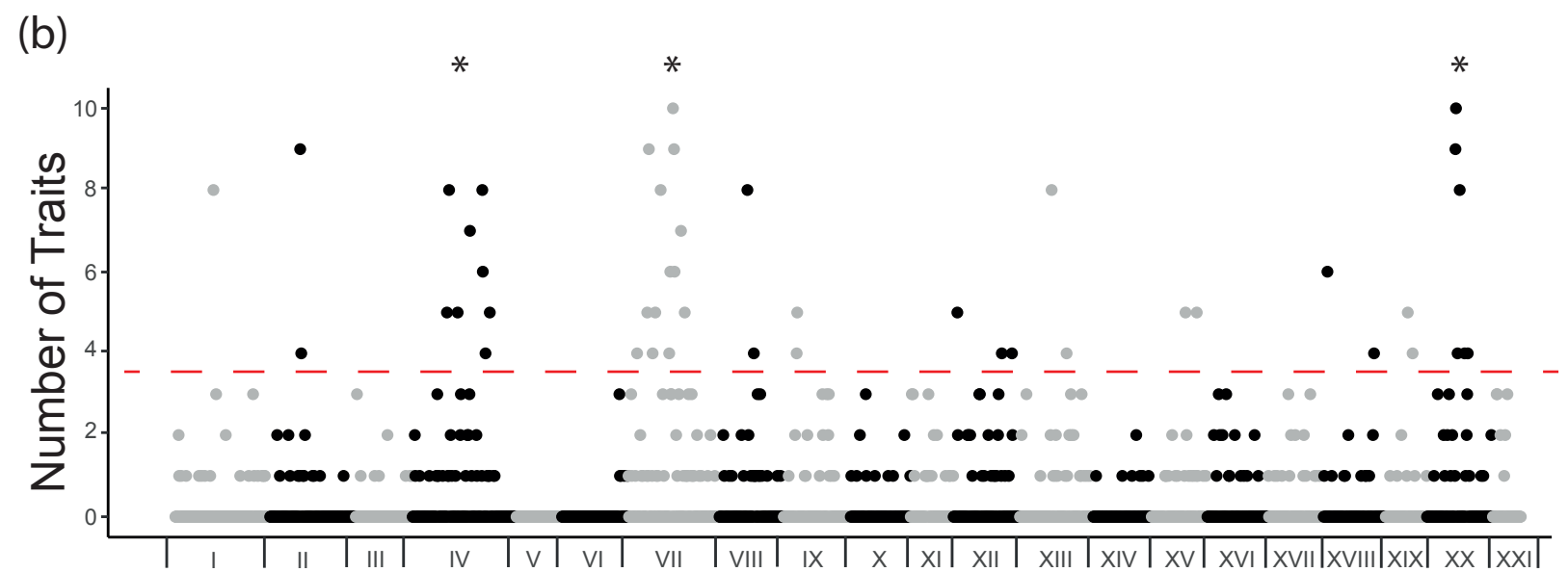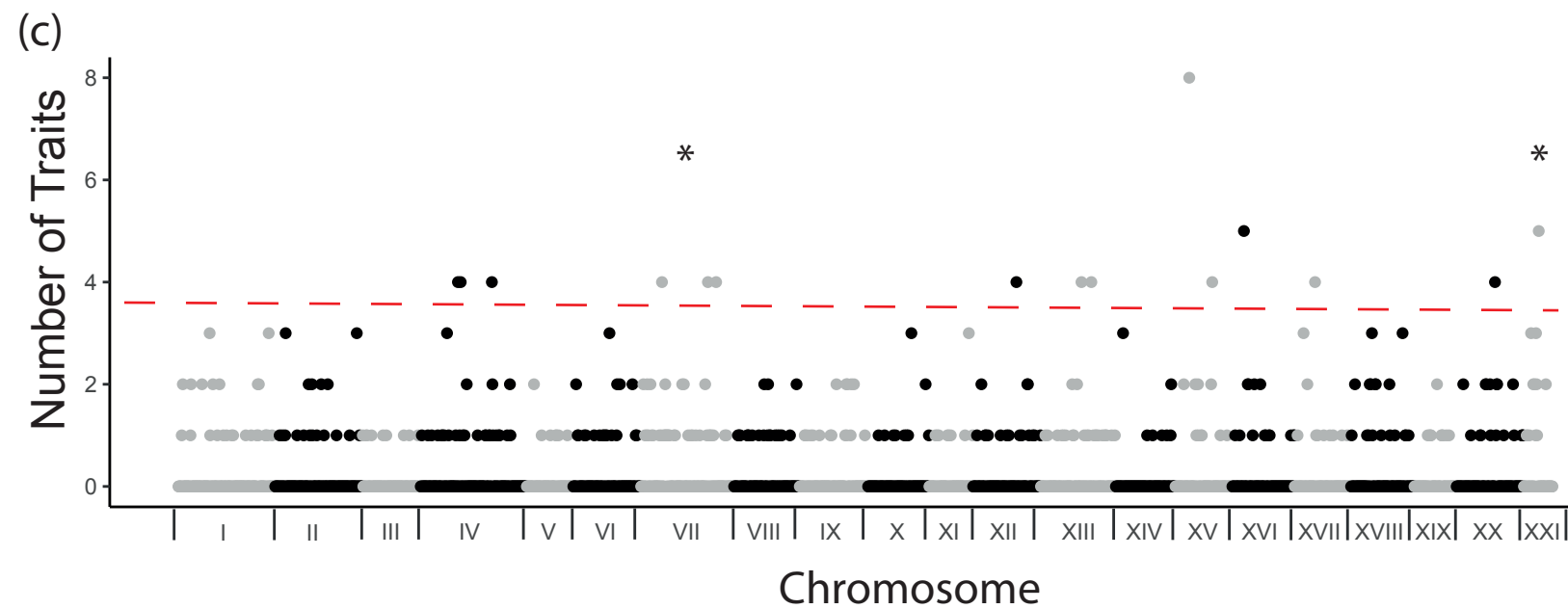

Supplement: Supplementary Figure 2 [file rstb20180241supp5.pdf]
